# The safety and efficacy of long-term use of calcitonin analogs in the treatment of osteoporosis in the elderly: a pharmacovigilance and RCT meta-analysis

**DOI:** 10.3389/fphar.2025.1514387

**Published:** 2025-09-29

**Authors:** Lingjie Tan, Bin Sheng, Sui Deng

**Affiliations:** ^1^ Department of Orthopedics (Bone Ward 7), Hunan Provincial People’s Hospital (The First Affiliated Hospital of Hunan Normal University), Changsha, China; ^2^ Changde Hospital, Xiangya School of Medicine, Central South University (The First People’s Hospital of Changde City), Changde, Hunan, China

**Keywords:** osteoporosis, calcitonin, FAERS database, pharmacovigilance, meta-analysis, fractures, bone mineral density

## Abstract

**Background:**

Osteoporosis is a common metabolic bone disease in the elderly, and its incidence continues to rise with the global aging population. Calcitonin analogs (including synthetic salmon, human, and porcine calcitonin preparations) are a classic treatment option for osteoporosis; however, the safety and efficacy of their long-term use remain controversial despite widespread application.

**Objective:**

This study aims to systematically assess the safety and efficacy of long-term use of calcitonin analogs in the treatment of osteoporosis in the elderly through pharmacovigilance analysis and meta-analysis.

**Methods:**

The study evaluated the long-term effectiveness and adverse effects of calcitonin analogs using pharmacovigilance data from the FAERS database and a meta-analysis of randomized controlled trials (RCTs). The pharmacovigilance analysis included adverse event data from osteoporosis patients aged 65 and older from 2004 to 2023, and signal detection was performed using the reporting odds ratio (ROR), proportional reporting ratio (PRR), and Bayesian confidence propagation neural network (BCPNN) methods. The meta-analysis included RCT studies related to calcitonin published after 2010, and a random-effects model was used to calculate the hazard ratio (HR) with a 95% confidence interval.

**Results:**

Pharmacovigilance analysis revealed that nasal discomfort (ROR = 283.4, PRR = 264.5, IC = 7.3, IC_025_ = 6.8) and abnormal product odor (ROR = 206.2, PRR = 201.9, IC = 7.1, IC_025_ = 6.1) were the most significant adverse reactions associated with calcitonin. Meta-analysis results showed no significant effect of calcitonin analogs in preventing new non-vertebral fractures and vertebral fractures (HR = 0.97, 95% CI: 0.76–1.24; HR = 0.93, 95% CI: 0.77–1.14). Changes in lumbar spine and femoral neck bone mineral density showed a slight upward trend but were not statistically significant. The analysis of NTx-1 levels (N-terminal telopeptide of type I collagen, a marker of bone resorption) revealed substantial heterogeneity, with significant variation in results across studies.

**Conclusion:**

Long-term use of calcitonin analogs for the treatment of osteoporosis in the elderly does not confer additional benefits and instead increases the risk of adverse reactions.

## Highlights

• Long-term use of calcitonin analogs shows limited efficacy in fracture prevention and bone density improvement, with notable adverse reactions, particularly from nasal spray formulations.

## 1 Background

Osteoporosis is one of the most common metabolic bone diseases in the elderly, characterized by reduced bone mass and deterioration of bone microarchitecture, leading to increased bone fragility and a significantly higher risk of fractures (Munoz et al., 2020; Armas and Recker, 2012). With the intensifying trend of global population aging, the prevalence of osteoporosis continues to rise. Meta-analysis results indicate that the global prevalence of osteoporosis is 18.3% (95% CI 16.2–20.7), with a prevalence of 23.1% (95% CI 19.8–26.9) in women and 11.7% (95% CI 9.6–14.1) in men (Salari et al., 2021). Osteoporosis-related hip fractures result in an approximate mortality rate of 8% in men and 3% in women over the age of 50 ^4^. Additionally, osteoporosis-related fractures impose a significant economic burden on society, costing approximately 17.9 billion USD annually in the United States and 4 billion GBP annually in the United Kingdom (Clynes et al., 2020). Treatment strategies for osteoporosis are categorized into anabolic and catabolic approaches, each targeting different aspects of bone metabolism (Kuril et al., 2024). Although a variety of treatment options exist, including bisphosphonates, selective estrogen receptor modulators (SERMs), calcitonin analogs, and bone formation promoters (Hernlund et al., 2013), the long-term safety and efficacy of these treatments remain a critical issue in clinical practice.

Calcitonin analogs have been widely recognized as a classic therapeutic option for osteoporosis since the 1980s (Mehta et al., 2003; McLaughlin et al., 2024). In a real-world study, calcitonin accounted for 90.5% of all anti-osteoporotic medications prescribed (Wang et al., 2020). Calcitonin reduces bone resorption by inhibiting osteoclast activity, thereby decreasing bone loss and playing a crucial role in preventing osteoporotic fractures (Gennari and Agnusdei, 1994; Bandeira et al., 2016). Systematic reviews have also shown that elcatonin, a form of calcitonin, can significantly reduce pain scores in patients with osteoporosis (Chen et al., 2019). However, in recent years, concerns have emerged regarding the potential risks associated with the long-term use of calcitonin, particularly its possible link to certain cancers, raising doubts about the safety of this treatment option (Wells et al., 2016; Sun et al., 2014; Srinivasan et al., 2020; Tu et al., 2023; Okamoto et al., 2020). The 2023 edition of the China guideline for diagnosis and treatment of senile osteoporosis (2023) recommends using calcitonin analogs in elderly patients with osteoporosis to alleviate pain, prevent rapid bone loss, and promote fracture healing, with a suggested treatment duration of no more than 3 months (Osteoporosis et al., 2023). However, the level of evidence supporting this recommendation is relatively low (Grade 2C). Thus, significant uncertainty remains regarding the long-term efficacy and safety of calcitonin in fracture prevention, highlighting the need for a more systematic and rigorous evaluation of its long-term use. This study aims to comprehensively assess the safety and efficacy of long-term calcitonin use in the treatment of osteoporosis in the elderly by combining pharmacovigilance data from the FDA Adverse Event Reporting System (FAERS) and results from a meta-analysis. The integration of pharmacovigilance data from FAERS with meta-analysis provides complementary insights: real-world safety signals from spontaneous reports, and efficacy data from controlled trials. This approach allows for a more comprehensive understanding of the benefit-risk profile of calcitonin analogs.

## 2 Methods

### 2.1 Study design

This study consisted of pharmacovigilance analysis based on the FDA Adverse Event Reporting System (FAERS) database and a meta-analysis of randomized controlled trials (RCTs) aimed at comprehensively evaluating the safety and efficacy of long-term use of calcitonin analogs in the treatment of osteoporosis in the elderly.

### 2.2 Data sources

The pharmacovigilance data were obtained from the FDA Adverse Event Reporting System (FAERS) and accessed via AERSMine (https://research.cchmc.org/aers/), a validated online platform that provides curated FAERS data. AERSMine applies the FDA’s recommended de-duplication procedures, using Individual Safety Reports (ISRs), Case IDs, and version numbers to ensure unique case identification. We selected data from the first quarter of 2004 to the third quarter of 2023 and extracted pharmacovigilance information related to calcitonin analogs, including calcitonin (salmon synthetic), calcitonin (human synthetic), and calcitonin (pork natural). Only records in which these agents were designated as the primary suspect drug were included in the analysis.

For the meta-analysis, we systematically searched PubMed, Embase, and the Cochrane Library databases using the keywords “calcitonin” and “osteoporosis.” We included only RCT studies published after 2010. Studies published after 2010 were selected to ensure the inclusion of recent trials with improved methodological quality, standardized reporting of outcomes, and greater clinical relevance to current treatment practices.Studies were eligible if they: (1) included elderly or postmenopausal osteoporosis patients; (2) evaluated long-term calcitonin treatment (≥3 months); and (3) reported efficacy or safety outcomes of interest. Studies were excluded if they: (1) evaluated calcitonin use <3 months; (2) had a sample size <100 participants; (3) enrolled non-osteoporotic populations; (4) were non-RCTs; or (5) lacked sufficient outcome data or failed to meet methodological quality standards.

### 2.3 Data extraction

Data extraction and analysis from FAERS were performed using the AERSmine platform. The target population of this study consisted of osteoporosis patients aged 65 years and older. All relevant adverse event reports were extracted, and specific adverse events related to calcitonin with six or more cases were analyzed in detail. The drug keywords used included “calcitonin preparations,” “calcitonin (salmon synthetic),” “calcitonin (human synthetic),” and “calcitonin (pork natural),” with the drug role limited to “primary suspect.”

For the meta-analysis, two independent researchers extracted study characteristics (author, year, sample size, baseline demographics, intervention, comparator, and primary outcomes). Risk of bias was assessed using the Cochrane Risk of Bias Tool, version 1 (RoB 1.0), and disagreements were resolved by discussion or a third reviewer. Study heterogeneity was quantified using the *I* ^*2*^ statistic.

### 2.4 Statistical analysis

Data analysis was performed using R software (version 4.2.3). Three widely used disproportionality analysis methods—the Reporting Odds Ratio (ROR), the Proportional Reporting Ratio (PRR), and the Bayesian Confidence Propagation Neural Network (BCPNN)—were employed to quantify the strength of the association between calcitonin analogs and specific adverse events. For each adverse event signal, RORs and PRRs with 95% confidence intervals (CIs) were estimated, and the BCPNN model provided information component (IC) values along with their lower 95% credibility bounds (IC_025_). A signal was considered positive when predefined criteria were met: for ROR, the lower limit of the 95% CI exceeded 1 with at least three cases (a ≥3); for PRR, values met the thresholds of PRR ≥2, a ≥3; and for BCPNN, IC_025_ was greater than 0, a > 0. Here, “a” represents the number of reports of the target adverse event associated with the drug of interest. The detailed calculation formulas and thresholds used for signal detection are provided in Supplementary Tables S1, S2.

For the meta-analysis, pooled hazard ratio (HR) with 95% CIs were estimated using a random-effects model, and forest plots were generated to visualize the results.

### 2.5 Ethical considerations

This study is based on secondary data analysis of publicly available data and does not involve direct research on individual patients. All data were anonymized, so no ethics committee approval was required.

## 3 Results

### 3.1 Basic characteristics of pharmacovigilance analysis

From the first quarter of 2004 to the third quarter of 2023, a total of 20,346,289 reports were submitted, of which 115,362 cases involved osteoporosis patients aged 65 and older. Among these, there were 1,333 reports of adverse reactions associated with calcitonin treatment, with 285 cases identified where calcitonin was the primary suspected drug (Table 1). The majority of patients in these adverse reaction reports were female (93.7%). The primary reporters were physicians and consumers. Additionally, the highest number of cases was reported between 2004 and 2008, after which the number of reports gradually declined. Regarding patient outcomes, 10 cases (3.5%) resulted in death, and 70 cases (24.6%) involved hospitalization or prolonged hospital stays due to calcitonin-related adverse reactions (Table 1).

**TABLE 1:** Basic characteristics of adverse events primarily suspected to be associated with calcitonin therapy in the elderly with osteoporosis.

Characteristic	Number of cases (%)
all AEs with calcitonin	1,333
Primary Suspect	285
Gender	
males	17 (6.0)
females	267 (93.7)
not reported	1 (0.4)
Reporter occupation	0 (0.0)
physician	64 (22.5)
pharmacist	13 (4.6)
Other healthcare professional	32 (11.2)
lawyer	0 (0.0)
consumer	96 (33.7)
sales	0 (0.0)
not reported	80 (28.1)
Reporting year	
2004–2008	162 (56.8)
2009–2013	72 (25.3)
2014–2018	33 (11.6)
2019–2023Q3	18 (6.3)
Outcomes	
died	10 (3.5)
disabled	12 (4.2)
hospitalized	70 (24.6)
life threatening	11 (3.9)
other outcomes	181 (63.5)
required intervention	6 (2.1)

### 3.2 Risk signal mining results

Table 2 shows the results of risk signal detection. This study identified nasal discomfort (ROR = 283.4, PRR = 264.5, IC = 7.3, IC_025_ = 6.8) and abnormal product odor (ROR = 206.2, PRR = 201.9, IC = 7.1, IC_025_ = 6.1) as the most significant adverse events associated with the use of calcitonin. Additionally, nasal bleeding (ROR = 21.3, PRR = 19.8, IC = 4.2, IC_025_ = 3.6), flushing (ROR = 21.4, PRR = 20.3, IC = 4.3, IC_025_ = 3.6), and rhinorrhea (ROR = 14.6, PRR = 13.9, IC = 3.8, IC_025_ = 3.0) were found to have a strong association. Adverse events related to the respiratory and cardiovascular systems, such as pulmonary edema (ROR = 9.0, PRR = 8.8, IC = 3.1, IC_025_ = 2.1) and chest discomfort (ROR = 7.4, PRR = 7.1, IC = 2.8, IC_025_ = 2.0), also exhibited certain risks. While events like dizziness and nausea were frequently reported, their association strength was lower (ROR <3.0, IC_025_ close to 0). Several adverse events (e.g., headache, back pain, rash) did not reach the threshold for signal detection by either PRR or BCPNN, suggesting no robust association.

**TABLE 2:** Primary suspected adverse events related to calcitonin in the elderly with osteoporosis.

Adverse events	Number of cases	ROR (95% CI)	PRR (95% CI)	IC	IC_025_
nasal discomfort	19	283.4 (156.9–511.6)	264.5 (150.1–466.2)	7.3	6.8
product odour abnormal	6	206.2 (76.9–553.3)	201.9 (76.3–534.2)	7.1	6.1
epistaxis	20	21.3 (13.4–33.8)	19.8 (12.9–30.6)	4.2	3.6
flushing	15	21.4 (12.6–36.4)	20.3 (12.3–33.7)	4.3	3.6
rhinorrhoea	15	14.6 (8.6–24.8)	13.9 (8.4–23.0)	3.8	3.0
pulmonary oedema	8	9.0 (4.4–18.3)	8.8 (4.4–17.5)	3.1	2.1
chest discomfort	13	7.4 (4.2–13.0)	7.1 (4.2–12.1)	2.8	2.0
deafness	6	7.2 (3.2–16.2)	7.0 (3.2–15.6)	2.8	1.6
device failure	6	5.6 (2.5–12.6)	5.5 (2.5–12.2)	2.4	1.3
blood pressure increased	15	3.9 (2.3–6.5)	3.7 (2.3–6.1)	1.9	1.2
vision blurred	9	4.4 (2.3–8.6)	4.3 (2.3–8.2)	2.1	1.2
blood glucose increased	6	4.1 (1.8–9.3)	4.1 (1.8–9.0)	2.0	0.9
pleural effusion	6	4.0 (1.8–8.9)	3.9 (1.8–8.6)	1.9	0.8
haemoglobin decreased	6	3.2 (1.4–7.1)	3.1 (1.4–6.9)	1.6	0.5
dizziness	31	2.0 (1.4–2.9)	1.9 (1.3–2.6)	0.9	0.4
chills	8	2.7 (1.3–5.5)	2.7 (1.3–5.3)	1.4	0.4
drug ineffective	13	2.3 (1.3–4.0)	2.3 (1.3–3.8)	1.2	0.4
cardiac disorder	7	2.5 (1.2–5.3)	2.4 (1.2–5.1)	1.3	0.2
nausea	28	1.7 (1.1–2.5)	1.6 (1.1–2.3)	0.7	0.2
back pain	23	1.6 (1.0–2.4)	1.5 (1.0–2.3)	0.6	0.0
vomiting	14	1.8 (1.0–3.0)	1.7 (1.0–2.9)	0.8	0.0
palpitations	7	2.1 (1.0–4.4)	2.1 (1.0–4.3)	1.0	0.0
headache	17	1.4 (0.8–2.3)	1.4 (0.9–2.2)	0.4	−0.2
spinal fracture	7	1.7 (0.8–3.5)	1.7 (0.8–3.4)	0.7	−0.4
dyspnoea	13	1.3 (0.8–2.3)	1.3 (0.8–2.2)	0.4	−0.4
rash	8	1.5 (0.8–3.1)	1.5 (0.8–3.0)	0.6	−0.4
arthralgia	23	1.2 (0.8–1.8)	1.1 (0.8–1.7)	0.2	−0.4
myocardial infarction	6	1.6 (0.7–3.7)	1.6 (0.7–3.6)	0.7	−0.5
insomnia	8	1.4 (0.7–2.8)	1.4 (0.7–2.8)	0.5	−0.5
pneumonia	11	1.2 (0.7–2.2)	1.2 (0.7–2.1)	0.2	−0.6
cough	7	1.4 (0.6–2.9)	1.4 (0.6–2.8)	0.4	−0.6
depression	8	1.3 (0.6–2.6)	1.3 (0.6–2.5)	0.4	−0.6
pyrexia	9	1.2 (0.6–2.4)	1.2 (0.6–2.3)	0.3	−0.6
atrial fibrillation	6	1.4 (0.6–3.1)	1.4 (0.6–3.1)	0.5	−0.7
pharmaceutical product complaint	6	1.3 (0.6–3.0)	1.3 (0.6–2.9)	0.4	−0.8
product complaint	6	1.3 (0.6–3.0)	1.3 (0.6–2.9)	0.4	−0.8
pruritus	6	1.1 (0.5–2.5)	1.1 (0.5–2.5)	0.2	−1.0
asthenia	12	0.9 (0.5–1.6)	0.9 (0.5–1.6)	−0.2	−1.0
weight decreased	7	0.9 (0.4–1.9)	0.9 (0.4–1.9)	−0.1	−1.2
pain	12	0.6 (0.4–1.1)	0.6 (0.4–1.1)	−0.6	−1.4
hypertension	6	0.8 (0.3–1.7)	0.8 (0.3–1.7)	−0.4	−1.5
gait disturbance	6	0.7 (0.3–1.6)	0.7 (0.3–1.6)	−0.5	−1.7
fatigue	8	0.5 (0.3–1.1)	0.5 (0.3–1.1)	−0.9	−1.9
fall	12	0.4 (0.2–0.7)	0.4 (0.2–0.7)	−1.3	−2.1

“Product odor abnormal” and “device failure” are non-clinical adverse events related to product quality or technical issues, and are presented here for completeness but should be distinguished from clinical adverse events.

### 3.3 Basic characteristics of included studies and bias assessment in the meta-analysis

After systematically searching the Embase, Cochrane, and PubMed databases, we initially identified 446 articles: 216 from Embase, 207 from Cochrane, and 23 from PubMed. Based on the inclusion and exclusion criteria, six RCTs (Henriksen et al., 2016; Binkley et al., 2012; Iwamoto et al., 2011; Li et al., 2013; Sugimoto et al., 2019; Tanaka et al., 2017) were ultimately included in the meta-analysis, involving a total of 8,653 patients, with a mean age range of 65.1–79.8 years. The duration of calcitonin treatment ranged from 6 to 36 months, and various administration routes were used, including oral, nasal spray, and intramuscular injection. The basic characteristics of the included studies are shown in Table 3. The study selection process is shown in Supplementary Figure S1.

**TABLE 3:** Basic characteristics of the literature included in the meta-analysis.

Study	Study population	Age (mean ± SD)	Case number	Intervention time (month)	Number of cases in the experimental group	Number of cases in control group	calcitonin administration route	Type of calcitonin	Dosage of calcitonin
2016 K. Henriksen	postmenopausal women	66.8 ± 6.1	4,665	36	2,334	2,331	oral	oral calcitonin (SMC021)	0.8 mg/d
2012 N. Binkley	postmenopausal women	66.4 ± 7.4	565	12	182	104	nasal spray	synthetic salmon calcitonin (ssCT)	200 IU/d
2011 J. Iwamoto	postmenopausal women	79.8 ± 6.9	194	6	96	97	intramuscular	Intramuscular elcatonin (ECT)	20 units a week
2013 Y. Li	postmenopausal women	65.1 ± 7.5	453	12	112	341	intramuscular	elcatonin	20 units a week
2019 T. Sugimoto	postmenopausal women	75.5 ± 5.7	869	36	433	436	intramuscular	elcatonin	20 units a week
2017 S. Tanaka	female patients ≥55 years old	74.6 ± 5.4	107	6	53	54	intramuscular	elcatonin	20 units a week

Among the six studies, three employed a double-blind design, indicating a lower risk of performance bias, while the other three studies were open-label designs, resulting in higher risks of performance and detection bias. All studies demonstrated good reporting bias, with no significant issues related to selective reporting identified. Attrition bias was generally low, and most studies used intention-to-treat (ITT) analysis to address loss to follow-up. The bias assessment of the included studies is summarized in Supplementary Table S3.

### 3.4 Meta-analysis results

#### 3.4.1 New fractures

**FIGURE 1:**
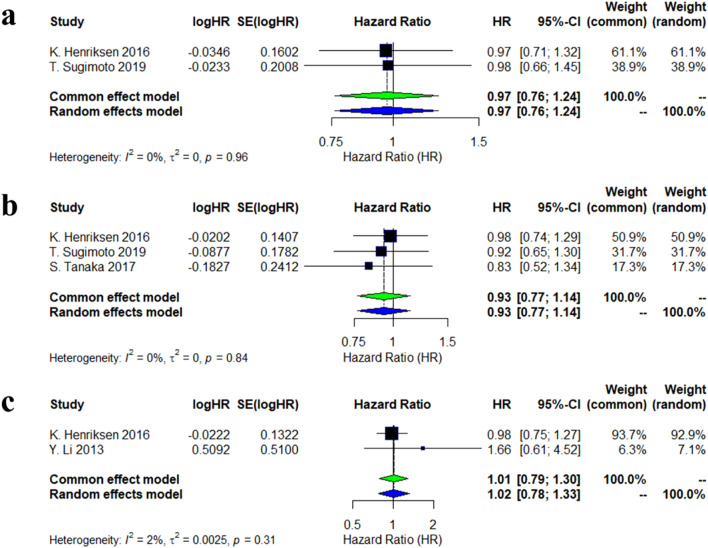
Forest plots of the estimation of incidences of new fracture. **(a)** new nonvertebral fractures; **(b)** new vertebral fracture; **(c)** new clinical fracture.

Two studies evaluated the risk of new non-vertebral fractures associated with calcitonin analogs. The meta-analysis showed no significant association between calcitonin use and the risk of new non-vertebral fractures (HR = 0.97, 95% CI: 0.76–1.24), with low heterogeneity between studies (I^2^ = 0%, p = 0.96). Three studies assessed the risk of new vertebral fractures, and the combined results similarly did not reveal a significant effect (HR = 0.93, 95% CI: 0.77–1.14), also with low heterogeneity (I^2^ = 0%, p = 0.84). For new clinical fractures, two studies indicated that calcitonin had no significant effect on the associated risk (Figure 1).

#### 3.4.2 Changes in bone mineral density (BMD)

**FIGURE 2:**
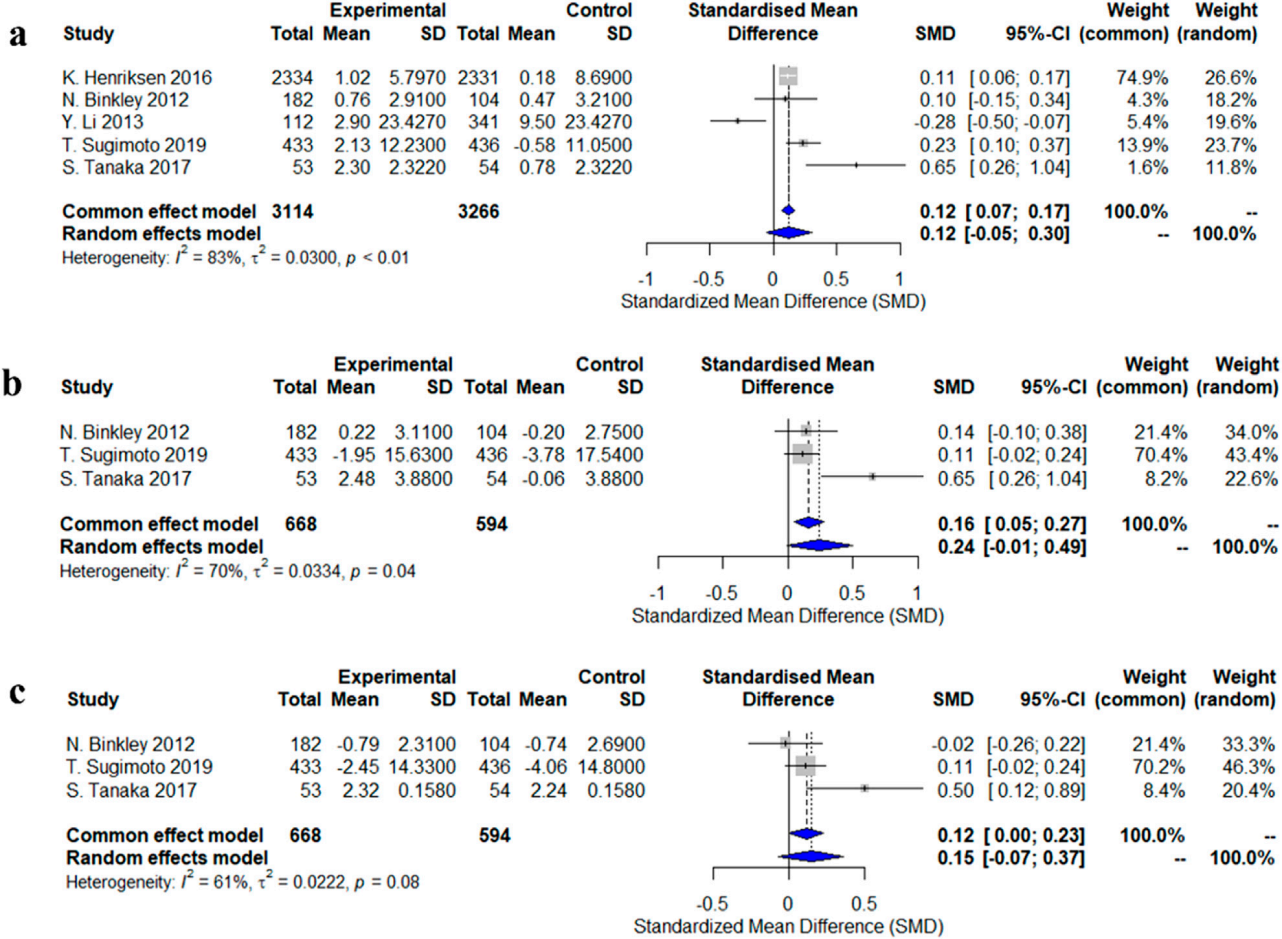
Forest plots of the estimation of changes in bone density. **(a)** lumbar spine; **(b)** femoral neck; **(c)** total hip.

Five studies evaluated changes in lumbar spine bone mineral density (BMD). The standardized mean difference (SMD) from the random-effects model was 0.12 (95% CI: −0.05–0.30), indicating a small and statistically non-significant effect of calcitonin analogs on lumbar spine BMD, with high heterogeneity (I^2^ = 83%, p < 0.01). Three studies assessed changes in femoral neck BMD, and the random-effects model showed an increasing trend (SMD = 0.24, 95% CI: −0.01–0.49), but this did not reach statistical significance, with moderate heterogeneity (I^2^ = 70%, p = 0.04). Some individual studies, such as Tanaka et al. (2017), reported a significant increase (SMD = 0.65, 95% CI: 0.26–1.04). For total hip BMD, a pooled analysis of three studies did not show statistical significance (SMD = 0.15, 95% CI: −0.07–0.37), with moderate heterogeneity (I^2^ = 61%, p = 0.08). Notably, Tanaka et al. (2017) reported a significant increase (SMD = 0.50, 95% CI: 0.12–0.89) (Figure 2).

#### 3.4.3 Changes in serum biomarkers

**FIGURE 3:**
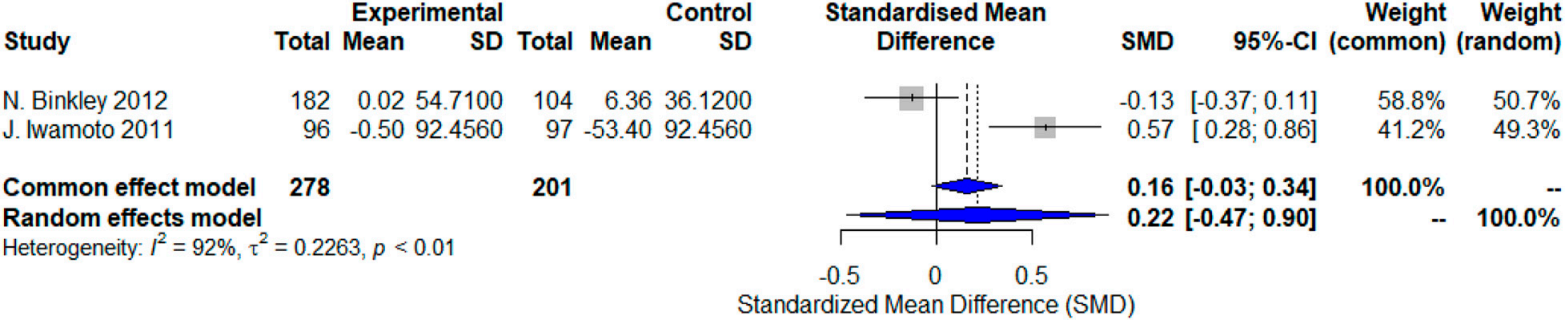
Forest plots of the estimation of changes in NTx-1.

Two studies evaluated changes in NTx-1 levels. The random-effects model analysis indicated a trend toward an increase in NTx-1 levels with the use of calcitonin analogs, but this was not statistically significant (SMD = 0.22, 95% CI: -0.47–0.90), with high heterogeneity (I^2^ = 92%, p < 0.01). There was significant variability in the study results: Binkley et al. (2012) reported a decreasing trend in NTx-1 levels (SMD = −0.13, 95% CI: -0.37 to 0.11), while Iwamoto et al. (2011) found a significant increase in NTx-1 levels (SMD = 0.57, 95% CI: 0.28–0.86) (Figure 3).

#### 3.4.4 Adverse reactions

**FIGURE 4:**
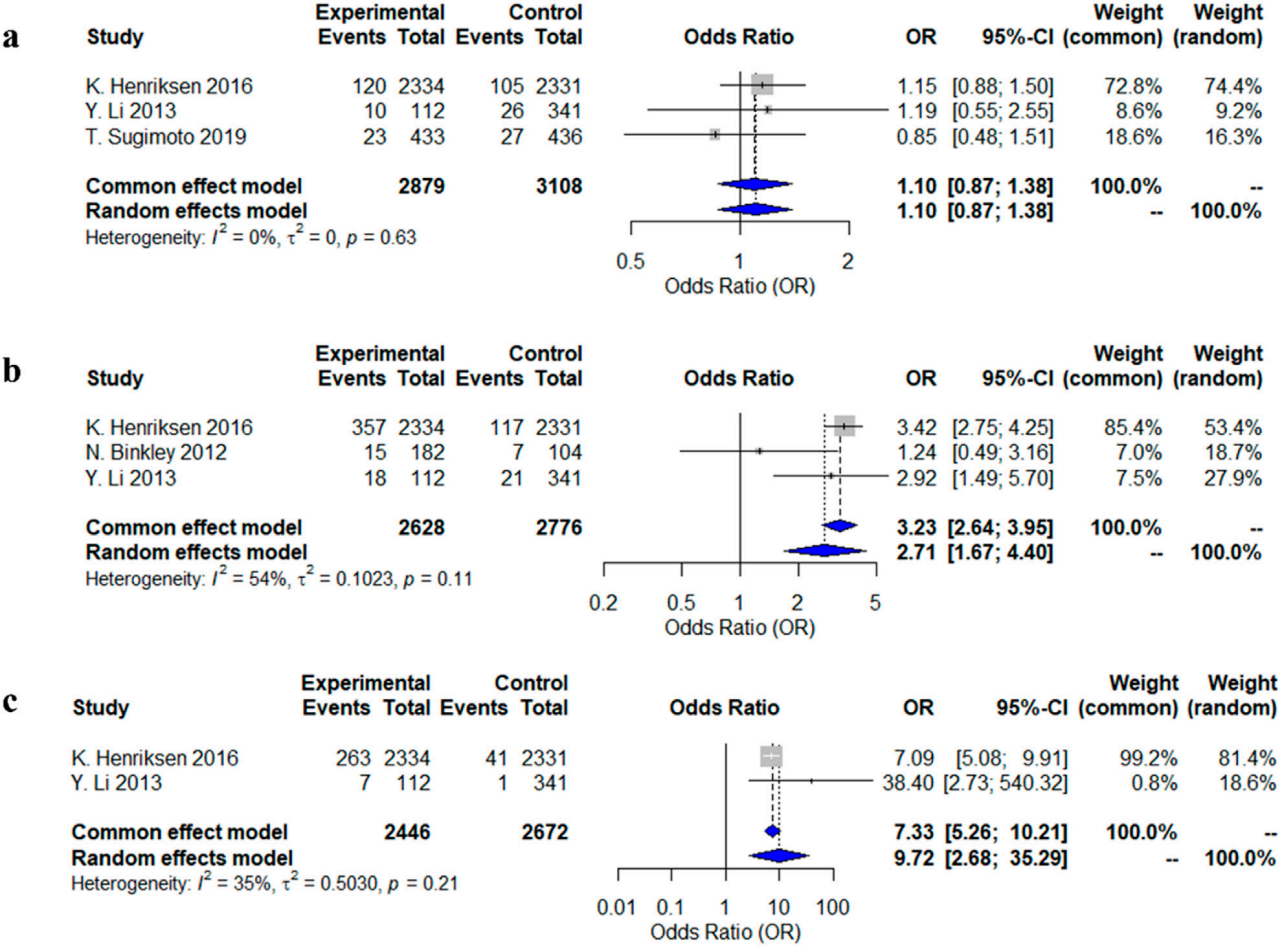
Forest plots of the estimation of adverse reactions. **(a)** dizziness; **(b)** nausea; **(c)** hot flushes.

Three studies reported dizziness and nausea as adverse reactions, and two studies reported flushing. The meta-analysis using a random-effects model showed that patients who used calcitonin for the long term were more likely to experience flushing and nausea compared to those who did not use calcitonin, with odds ratios (OR) and 95% confidence intervals (CI) of 2.71 (1.67–4.40) and 9.72 (2.68–35.29), respectively. However, there was no significant difference in the risk of dizziness between the two groups (OR = 1.10, 95% CI: 0.87–1.38) (Figure 4).

## 4 Discussion

This study comprehensively evaluated the safety and efficacy of long-term use of calcitonin analogs in the treatment of osteoporosis in the elderly by combining pharmacovigilance analysis from the FAERS database with a meta-analysis of randomized controlled trials. The results revealed that the effects of these drugs on fracture prevention and bone mineral density improvement are limited, while highlighting their potential association with various adverse reactions during long-term use.

Although our results indicate limited long-term efficacy of calcitonin in increasing bone mineral density and reducing fracture risk, its initial FDA approval in the 1980s was based on early clinical evidence demonstrating anti-resorptive effects and pain relief in postmenopausal women (Mehta et al., 2003). At that time, few alternative therapies were available, and calcitonin was considered a relatively safe option. However, with growing evidence of limited effectiveness and potential cancer risks, regulatory authorities have since updated their guidance. The FDA now restricts calcitonin use primarily to short-term treatment or for patients who cannot tolerate other osteoporosis drugs. Our findings are consistent with this evolving regulatory perspective and underscore the importance of ongoing benefit-risk assessments in clinical practice.

The individual variation in the efficacy of calcitonin may be related to the baseline severity of osteoporosis, bone mineral density, and the route of administration. The absorption rate of the nasal spray form is relatively low, which may lead to insufficient therapeutic effects. In contrast, long-term and targeted calcitonin delivery systems, developed using advanced drug delivery strategies such as micro/nano-drugs, gels, prodrugs, and composite biomaterials, may overcome the limitations of traditional calcitonin. These systems hold promise for treating hypercalcemia, osteoporosis, and arthritis (Yu et al., 2021). Studies have suggested that combining calcitonin with alfacalcidol in osteoporosis treatment can be beneficial, effectively improving bone metabolism markers, increasing bone density, alleviating symptoms, enhancing quality of life, and reducing inflammation levels (Rui et al., 2024). It is noteworthy that the efficacy of calcitonin in inhibiting bone resorption is lower than that of other treatments, such as bisphosphonates and antibody-based drugs, and there are individual differences in response to its effects. A meta-analysis evaluating the efficacy of different drugs in osteoporosis found that teriparatide, abaloparatide, denosumab, and romosozumab significantly reduced vertebral fractures, while ibandronate and selective estrogen receptor modulators showed lower efficacy (Murad, 2021). Additionally, parathyroid hormone is associated with a higher incidence of adverse events. Hormone replacement therapy (HRT) and calcitonin have slower onset of action (Wei et al., 2023).

Adverse reactions to calcitonin analogs, particularly when administered as a nasal spray, are concentrated in nasal discomfort, nosebleeds, and other related issues. These adverse effects may negatively impact treatment adherence in elderly patients. While our data did not directly measure adherence rates, prior studies have suggested that local irritation and discomfort are significant reasons for discontinuation of calcitonin therapy. This is particularly relevant in long-term management, where patient comfort and tolerability are essential for maintaining therapeutic continuity. Hence, minimizing adverse effects through alternative delivery methods, such as oral or transdermal routes, may improve adherence and overall treatment outcomes. In future clinical practice, oral (Bandeira et al., 2016) or transdermal administration (Li et al., 2023) may be more optimal choices. Li et al. (Li et al., 2023) developed a composite detachable microneedle system, offering a promising avenue for transdermal calcitonin delivery, which could reduce adverse effects and improve patient adherence. Recent advances in peptide engineering have enabled the development of stapled analogs of salmon calcitonin to overcome gastrointestinal degradation. For instance, a novel analog named KaY-1 (R24Q), stabilized via cooperative Lys–Tyr stapling, demonstrated significantly improved stability in simulated gastric and intestinal fluids while retaining full bioactivity at the calcitonin receptor (Ghareeb and Metanis, 2023).

In recent years, studies linking calcitonin analogs to certain cancers have raised widespread concern. For example, results from population-based nested case-control studies suggest that calcitonin use may increase the risk of liver cancer in female osteoporosis patients while reducing the risk of breast cancer (Sun et al., 2014). Moreover, calcitonin, as a growth-stimulating peptide derived from prostate epithelium, may promote prostate cancer progression through paracrine factors (Chien et al., 2001). Although this study did not find a significant increase in cancer risk, the long-term safety of calcitonin analogs should be carefully considered in clinical settings, particularly for individuals with potential cancer risks.

This study has several limitations. First, the data from the FAERS database are based on voluntary reports, which may introduce reporting bias, particularly with minor adverse reactions potentially being underreported. Moreover, the absence of denominator data limits our ability to determine true incidence rates. Despite these limitations, FAERS remains a valuable tool for detecting safety signals, especially when triangulated with clinical trial data. Second, the number of RCTs included in the meta-analysis was limited, with study populations primarily consisting of postmenopausal women, and some studies had small sample sizes, which could affect the robustness of the results. High heterogeneity in some analyses may also influence the interpretation of the findings. Since the included studies did not report patients' concomitant medications or the specific treatment phase of calcitonin (e.g., whether it was first-line therapy), our analysis of adverse effects may be subject to potential bias.

Third, the analysis did not stratify the results by the type of calcitonin analog used, despite known differences in amino acid sequences, receptor binding affinities, and immunogenicity. For example, salmon calcitonin, commonly used in clinical formulations, consists of 32 amino acids and differs from human calcitonin by 16 amino acid residues, contributing to its higher receptor affinity and longer biological half-life. Porcine calcitonin is more structurally similar to human calcitonin than salmon calcitonin but exhibits intermediate potency.

Additionally, the role of individual differences in calcitonin efficacy has not been thoroughly explored. Future research should focus on the impact of individual characteristics, drug dosage, routes of administration and specific types of calcitonin analogs used.

## 5 Conclusion

This study systematically evaluated the long-term application of calcitonin analogs in the treatment of osteoporosis in the elderly, confirming the limited efficacy of these drugs in fracture prevention and bone mineral density improvement. Furthermore, calcitonin analogs are associated with various adverse reactions, with nasal spray formulations showing the most prominent side effects. Future clinical use should emphasize optimizing drug dosage, administration routes, and individualized treatment, combined with safety monitoring to reduce the potential risks of long-term use.

## Data Availability

The original contributions presented in the study are included in the article/Supplementary Material, further inquiries can be directed to the corresponding author.
