# Supplementary material for: The safety and efficacy of long-term use of calcitonin analogs in the treatment of osteoporosis in the elderly: a pharmacovigilance and RCT meta-analysis
Source: Front Pharmacol. 2025 Sep 29;16:1514387. doi: 10.3389/fphar.2025.1514387 (PMC12515915; doi:10.3389/fphar.2025.1514387)
Supplement: Supplementary file 1 [file DataSheet1.docx]

Supplementary Table 1 2×2 Contingency Table for Pharmacovigilance Signal Detection (ROR, PRR, BCPNN)

|  | target AE | other AEs | total |
| --- | --- | --- | --- |
| target drugs | a | b | a+b |
| other drugs | c | d | c+d |

ROR: Reporting Odds Ratio; PRR: Proportional Reporting Ratio; BCPNN: Bayesian Confidence Propagation Neural Network; AE: adverse events;

a: Number of cases of the target adverse event associated with the target drug.

b: Number of cases of other adverse events associated with the target drug.

c: Number of cases of the target adverse event associated with other drugs.

d: Number of cases of other adverse events associated with other drugs.

Supplementary Table 2 Methods for pharmacovigilance signal detection

| Method | Calculation | Signal Detection Criteria |
| --- | --- | --- |
| ROR | $ROR=\frac{\left( a/c \right)}{b/d}=\frac{ad}{bc}$  $95\% CI=e^{\ln\left( ROR \right)\pm1.96}\sqrt{\frac{1}{a}+\frac{1}{b}+\frac{1}{c}+\frac{1}{d}}$ | lower 95% CI ＞ 1, a≥3 |
| PRR | $PRR=\frac{a/\left( a+b \right)}{c/\left( c+d \right)}$  $\chi^{2}=\sum\frac{\left( O-E \right)^{2}}{E} O = a E=\frac{\left( a+b \right)\left( a+c \right)}{a+b+c+d}$  $95\% CI=e^{\left( \ln\left( PRR \right)\pm1.96\times\sqrt{\frac{1}{a}-\frac{1}{a+b}+\frac{1}{c}-\frac{1}{c+d}} \right)}$ | PRR ≥ 2, $\chi^{2}$≥ 4, a ≥ 3 |
| BCPNN | $IC=\log_{2} \frac{a\left( a+b+c+d \right)}{\left( a+b \right)\left( a+c \right)}$  $IC_{025}=IC-2\sqrt{V\left( IC \right)}$ | IC_025_＞ 0, a ＞ 0 |

Supplementary Table 3 Bias assessment of literature included in meta-analysis

| Study | Random Sequence Generation | Allocation Concealment | Blinding | Incomplete Outcome Data | Selective Reporting | Other Bias |
| --- | --- | --- | --- | --- | --- | --- |
| 2016 K. Henriksen | Low risk | Low risk | Low risk | Low risk | Low risk | Moderate risk |
| 2012 N. Binkley | Low risk | Low risk | Low risk | Moderate risk | Low risk | Moderate risk |
| 2011 J. Iwamoto | Unclear risk | Unclear risk | High risk | Low risk | Low risk | Moderate risk |
| 2013 Y. Li | Unclear risk | Unclear risk | High risk | Moderate risk | Low risk | Moderate risk |
| 2019 T. Sugimoto | Unclear risk | Low risk | High risk | Low risk | Low risk | Moderate risk |
| 2017 S. Tanaka | Unclear risk | Low risk | High risk | Low risk | Low risk | Moderate risk |


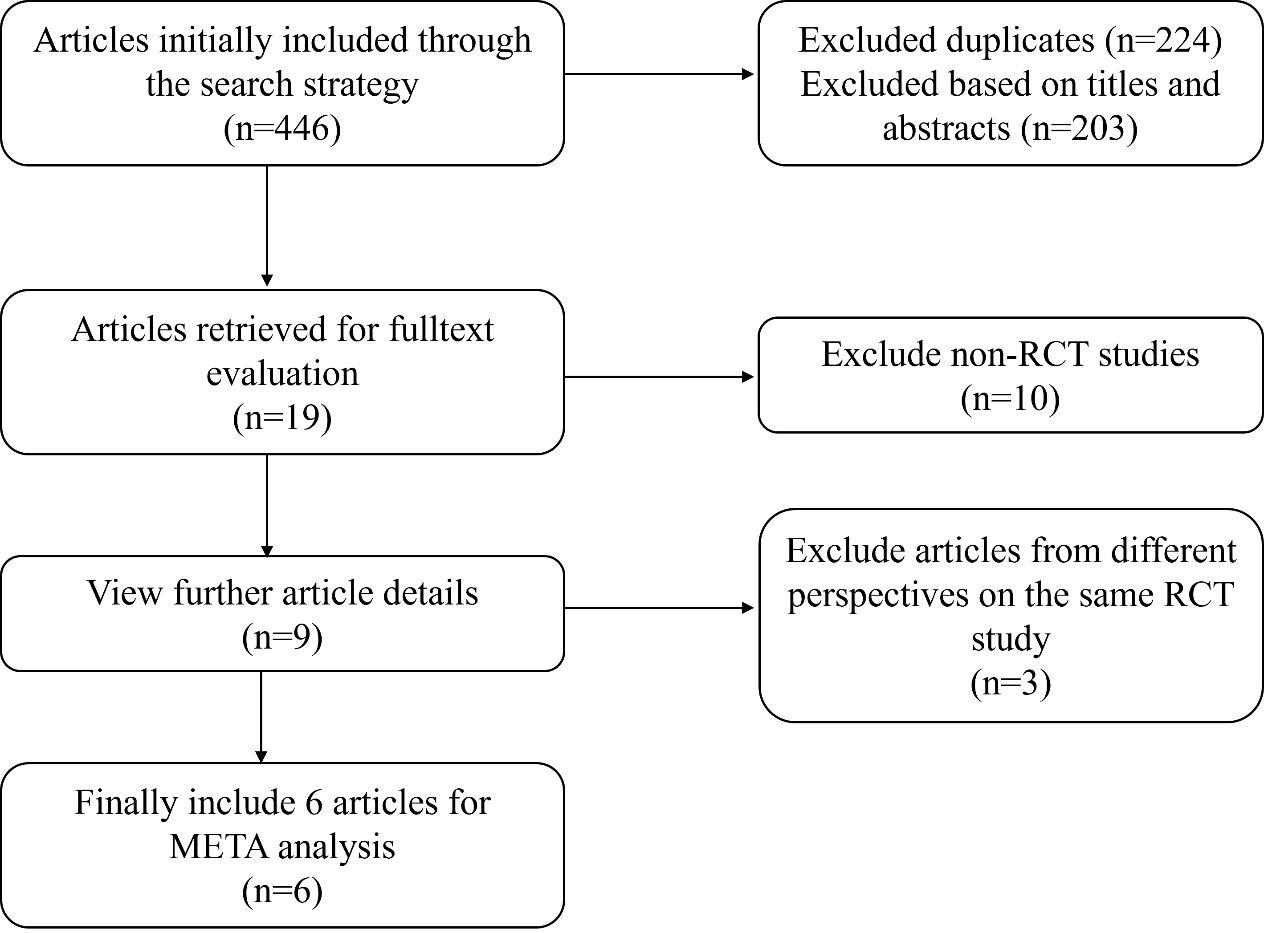
Supplementary Figure 1 Flow chart of literature inclusion and exclusion
